# Effectiveness of Lifestyle Modification in Polycystic Ovary Syndrome Patients with Obesity: A Systematic Review and Meta-Analysis

**DOI:** 10.3390/life12020308

**Published:** 2022-02-18

**Authors:** Chan-Hee Kim, Seon-Heui Lee

**Affiliations:** 1 Artificial Kidney Team, Incheon Sejong Hospital, Incheon 21080, Korea; potbeau@gmail.com; 2 Department of Nursing Science, College of Nursing, Gachon University, Incheon 21936, Korea

**Keywords:** polycystic ovary syndrome, obesity, lifestyle intervention, diet, exercise

## Abstract

(1) Background: Polycystic ovary syndrome (PCOS) is the most common cause of anovulatory infertility and endocrine disorders among women of reproductive age. Previous studies have employed lifestyle interventions to manage anovulatory infertility and endocrine disorders. However, the effect of lifestyle interventions on the metabolic index remains ambiguous; (2) Methods: Data were obtained through a systematic search of the Ovid-Medline, Ovid-EMBASE, and Cochrane Library databases. Two reviewers independently reviewed the literature in two stages. A consensus was achieved through discussions regarding the final selection of the literature; (3) Results: This study observed that the group that underwent lifestyle modifications displayed significant improvement in reproductive function compared to the control group. Combination therapy with diet and exercise resulted in improved fasting insulin levels, compared to monotherapy with diet or exercise. Moreover, moderate weight loss (a minimum of 5%) resulted in an improved metabolic index. The subgroup analysis revealed that the group that underwent lifestyle modifications had a significantly higher number of patients with improved menstrual cycles, compared to the control groups; (4) Conclusions: Lifestyle modification using combination therapy is a promising therapeutic approach that can be employed in the management of PCOS patients with obesity. This scenario warrants further studies with larger sample sizes to develop ideal treatment protocols.

## 1. Introduction

Polycystic ovary syndrome (PCOS) is the most common cause of anovulatory infertility and endocrine disorders and affects approximately 8–13% of reproductive age women [1,2]. The diagnosis and management of PCOS is a challenging endeavor because it is a mysterious condition with major symptoms that vary with age, and the treatment should be tailored to meet the specific requirements of each patient [3]. The application of the Rotterdam criteria for the diagnosis of adult women with PCOS was approved by international evidence-based guidelines. The diagnosis requires fulfillment of a minimum of two of the following three conditions: oligo-ovulation or anovulation, clinical or biochemical hyperandrogenism, and detection of the radiographic features of polycystic ovaries by means of ultrasonography [2]. The main symptoms of the syndrome include infertility attributable to anovulation, irregular menstrual cycles, and symptoms caused by androgen excess, such as hirsutism [4]. Moreover, the condition can be associated with concurrent chronic metabolic diseases, such as increased insulin resistance, which necessitates appropriate treatment to prevent complications [3].

Obesity is one of the most common concerns among patients with PCOS. In addition, there is a high correlation between obesity and the prevalence of PCOS. The prevalence of PCOS is 4.3% among women with a body mass index (BMI) less than or equal to 25 kg/m^2^ and 14% among women with a BMI above 30 kg/m^2^ [5]. Moreover, it has been reported that the risk of obesity is four times higher among patients with PCOS than among healthy controls [6]. Previous studies have shown that a high BMI causes metabolic abnormalities in patients with PCOS, such as increased insulin resistance and exacerbation of hyperandrogenemia [2]. Increased body weight and insulin resistance are the underlying causes of symptoms in PCOS patients with obesity. Hence, international evidence-based guidelines emphasize the importance of pre-pregnancy weight management among PCOS patients [2].

Lifestyle modification is recommended as the primary treatment for weight management in PCOS patients with obesity [3]. A variety of balanced dietary approaches to reduce dietary caloric intake and a gradual increase in physical activity are recommended to accomplish weight loss [2]. Several previous studies have endeavored to improve the lifestyles of patients with PCOS using various methods, such as diet, exercise therapy, and behavioral therapy. Consequently, a previous review reported that lifestyle modification programs were observed to affect weight loss or BMI among PCOS patients with or without obesity [7], and it is necessary to confirm the efficacy of lifestyle modification programs in the management of PCOS patients with obesity who struggle with weight management. Conversely, previous studies have reported ambiguous results regarding the variation in metabolic indicators after lifestyle interventions among patients with PCOS [7]. Furthermore, despite the fact that improvement in reproductive function is an important factor regarding PCOS, the effect of lifestyle interventions on the improvement of reproductive function has not been confirmed to date.

Hence, this study aimed to present an updated paper to confirm the effectiveness of lifestyle modification programs in the management of PCOS patients with obesity and confirm the effect of lifestyle modification programs on improving reproductive function for the first time. In addition, this study performed a subgroup analysis according to the type of intervention and degree of weight loss.

## 2. Materials and Methods

The research protocol of the present systematic review was registered with the National Research Foundation of Korea prior to the commencement of this research (No. 2020R1F1A1073141). The abovementioned protocol could not be modified arbitrarily, and the pre-selected patient, intervention, comparison, and outcome (PICO) could not be fixed by the researcher, which prevented selection reporting bias (List S1).

### 2.1. Data Sources and Search Strategy

The current review (Table S1) performed a systematic search of three databases to identify eligible articles, which are stated as follows: Ovid-Medline (1946 to 18 June 2021), Ovid-EMBASE (1974 to 18 June 2021), and Cochrane Library, according to the guidelines of the Cochrane community (https://community.cochrane.org/) (accessed on 18 June 2021). All papers published prior to the commencement of the literature search were analyzed. Moreover, additional documents were identified through hand-searching, and the references of selected documents (reference of reference) were searched. Relevant articles were included to supplement the comprehensiveness of the search. Keywords and Medical Subject Headings (MeSH) were used in combination to identify all relevant articles. The terms pertaining to the participants included “exp body mass index/,” “exp overweight/,” “exp obesity/,” “BMI,” “exp infertility/,” “exp anovulation/,” and “exp polycystic ovary syndrome/.” The terms pertaining to intervention included “lifestyle modification,” “lifestyle intervention,” “lifestyle change,” “lifestyle program,” “exp diet/,” “exp exercise/,” and “exp weight loss/.”

### 2.2. Eligibility Criteria and Selection Process

The articles included in the current review were selected by evaluating all the papers obtained through a literature search, in accordance with predefined inclusion and exclusion criteria. The inclusion criteria were as follows: (a) studies that involved patients diagnosed with PCOS and obesity, (b) studies that involved a lifestyle intervention program (diet and/or exercise program), and (c) studies that assessed more than one variable of interest (reproductive, anthropometric, androgenic, and metabolic indices). The present study excluded review articles, abstracts, conference posters, articles written in languages other than English, and duplicate publications.

In the current review, two reviewers independently selected and excluded papers in two stages. In the first stage of selection/exclusion, the documents that were deemed irrelevant to the present systematic review were excluded by screening the titles and abstracts according to the predefined criteria. In the second stage, full texts of the documents selected during the first stage were reviewed, and appropriate articles were selected. Consensus was achieved through discussions regarding the final selection of relevant literature. Reliability was assessed using Cohen’s kappa coefficient (κ = 0.83).

### 2.3. Variables and Data Collection

The reviewers performed data extraction using a prearranged data extraction form and double-checked the same. The variables that were documented are stated as follows: characteristics pertaining to the included participants and the type of intervention employed by the studies were recorded. In the current review, age, definition of obesity, and the criteria used to diagnose PCOS were recorded as the characteristics of the participants. Furthermore, the type of intervention; specific components of the intervention program; and duration, frequency, and time of day with reference to the implementation of intervention were recorded as the characteristics pertaining to the intervention program, that is, lifestyle modification.

In addition, the current review assessed the clinical outcomes of lifestyle modification programs. The variables of interest included reproductive, anthropometric, androgenic, and metabolic indices. The indices used in the studies were as follows: (a) reproductive index: number of patients with regular/irregular menstrual cycle, number of patients with improvement in menstrual cycle, ovarian volume, and number of ovarian follicles; (b) anthropometric index: weight, BMI, and waist circumference (WC); (c) metabolic index: fasting glucose level, fasting insulin level, and homeostatic model assessment for insulin resistance (HOMA-IR); (d) androgenic index: testosterone level, sex-hormone binding globulin (SHBG), and free androgen index (FAI).

### 2.4. Risk of Bias Assessment

In the current review, two independent reviewers assessed the methodological quality of the studies. Disagreements were resolved through consensus meetings. The tools used for the assessment of the risk of bias were contingent upon the study design. This study used the Cochrane Risk of Bias tool (RoB) and the Risk of Bias Assessment Tool for Non-randomized Studies (RoBANS) to evaluate randomized control trials (RCTs) and other studies. Each criterion in the tool was rated as follows: “low,” “high,” or “unclear” risk of bias.

### 2.5. Analysis

Data management and meta-analysis were performed using the RevMan program (Review Manager 5.4) for items that were reported by two or more studies. The current study computed the combined estimate of the odds ratios (ORs) pertaining to two groups using the Mantel–Haenszel method to assess dichotomous variables. The effect measures of continuous variables were estimated as mean difference (MD) and 95% confidence intervals (CI) using the inverse variance method. The chi-squared test was used to assess statistical heterogeneity among studies, and the significance was set at *p* < 0.10. Heterogeneity was quantified using the I2 statistic. In the absence of heterogeneity, the current meta-analysis employed a fixed-effects model as the basis for the statistical model. In the current review, a *p*-value less than 0.05 was considered to be statistically significant. Publication bias could not be evaluated owing to the inadequate number of studies included in the meta-analysis.

This study was approved by the Ethics Review Committee (1044396-202010-HR-192-01).

## 3. Results

**Figure 1:**
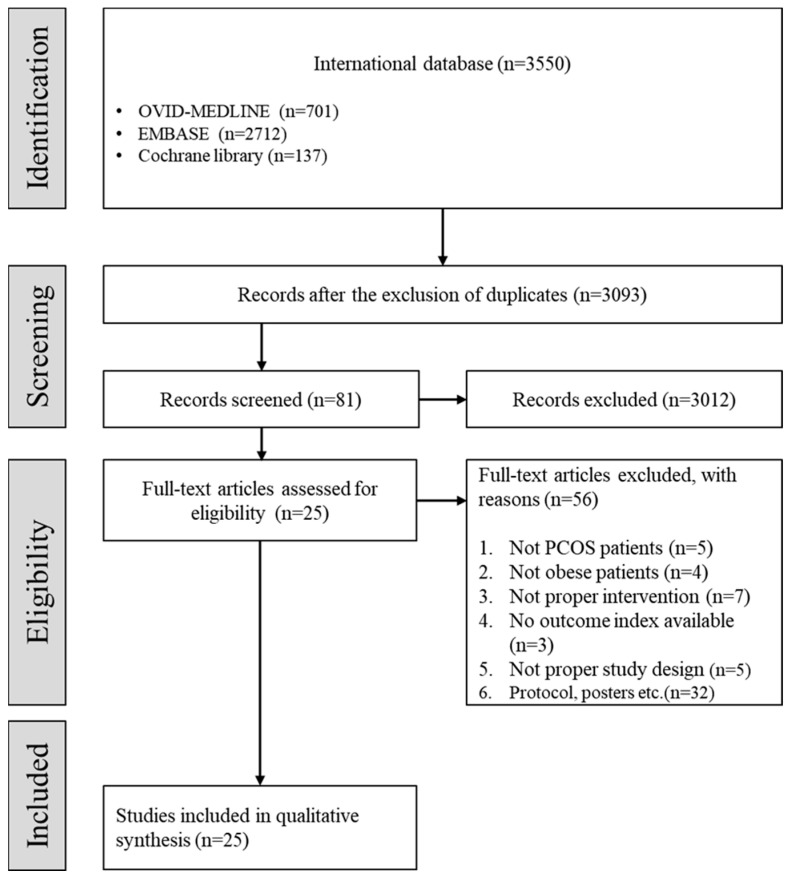
Flow chart depicting the selection of studies. (PCOS: polycystic ovary syndrome.)

Of the 3093 studies, 3012 studies were excluded by screening the titles and abstracts, followed by 56 studies from the full-text review. A total of 25 studies were included in the present systematic review after the selection process based on the aforementioned inclusion and exclusion criteria (Figure 1).

### 3.1. Study Characteristics

A summary of the characteristics of these studies is presented in Table 1. Among the 25 articles included in the current systematic review, eight were RCTs and 17 were non-randomized clinical trials or observational studies. Considering the geographic distribution, nine studies were published in Europe, seven in North America, five in Oceania, two in Asia, and one each in Africa and the Middle East.

All studies included in the current review involved overweight or obese PCOS patients. Regarding the inclusion criteria used by the studies, 21 included overweight or obese subjects who were included on the basis of the value of BMI, among which, ten, five, and three studies used BMI values of 25, 27, and 30, respectively. Additionally, one study used a BMI value of 28.6. Conversely, two studies presented data as age-specific percentiles. Moreover, only one study selected subjects with obesity based on body weight, whereas three studies did not state the specific criteria used to identify obesity.

Regarding the criteria used to diagnose PCOS, eight studies employed the Rotterdam diagnostic criteria, one study used the National Institutes of Health diagnostic criteria, and one study employed both. Among the studies included in the present review, five diagnosed PCOS on the basis of clinical characteristics, two employed radiographic evaluation by means of ultrasound scans, three studies used a combination of clinical characteristics and ultrasound scans, and five studies did not state the exact diagnostic criteria or methods used for diagnosis, although the subjects were reported to be PCOS patients.

The lifestyle modification program was divided into two groups: monotherapy with diet or exercise and combination therapy involving diet and exercise (hereinafter referred to as combination therapy). Regarding the type of intervention program used by the studies, nine used monotherapy with diet, six employed monotherapy with exercise, and ten studies used combination therapy (Table 2).

This study analyzed the reproductive, anthropometric, androgenic, and metabolic indices to confirm the effects of the lifestyle modification program in PCOS patients with obesity. Among the studies included in the present review, five [8,9,12,14,31] compared the data pertaining to the intervention group who received the lifestyle modification program and the control group who received usual or minimal care. Summary estimates of individual studies were synthesized by meta-analysis when the results of two or more studies were reported using the same subindex. Additionally, the statistical significance of the data pertaining to the 20 studies that did not compare the group that underwent the lifestyle intervention program with the control group was confirmed by comparing the results before and after the intervention.

**Figure 2:**
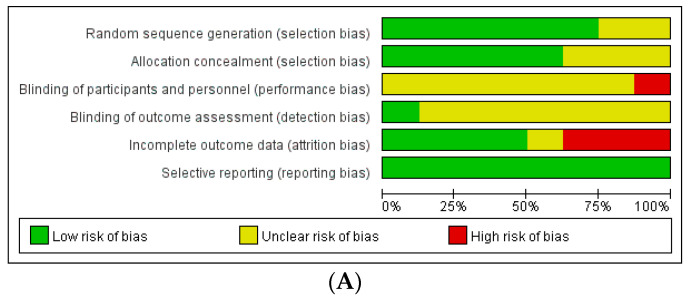

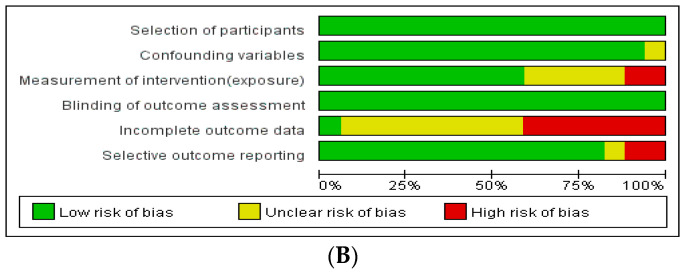
Assessment of risk of bias. (**A**) Cochrane Risk of Bias tool (RoB). (**B**) Risk of Bias Assessment Tool for Non-randomized Studies (RoBANS).

The assessment of the risk of bias in these studies revealed the following results (Figure 2): Among the RCTs, seven studies had a low risk of selection bias, and seven studies reported an unclear risk of blinding of participant, personnel, and outcome assessments. Three out of eight studies had a high risk of attrition bias associated with high drop rates. Among non-randomized studies, seven out of 17 studies had a high risk of attrition bias related to incomplete outcome data.

### 3.2. Effectiveness of Lifestyle Modification

#### 3.2.1. Reproductive Index

**Figure 3:**
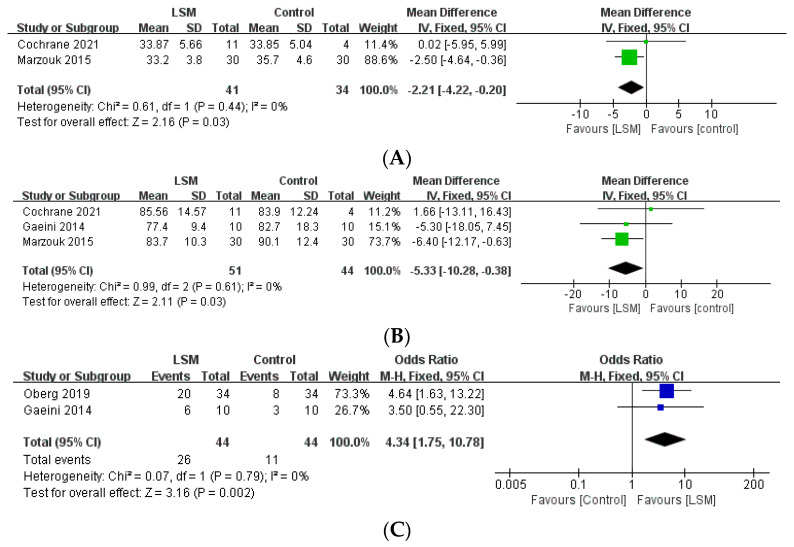
Forest plot for the meta-analysis of lifestyle modification, compared to controls. (**A**) BMI. (**B**) Weight. (**C**) Improved menstrual cycle. (CI: confidence interval, LSM: lifestyle modification, SD: standard deviation.)

Among the studies included in the present review, reproductive indices were used to assess the outcomes and report the results of 24 studies (Table 3). Among the aforementioned five studies that compared the intervention and control groups, the lifestyle modification group had a significantly higher number of patients with improved menstrual cycles, compared to the control group (OR: 4.34, 95% CI: 1.75–10.78, *p* = 0.02, I2 = 0%, Figure 3). Moreover, one study [12] reported that the intervention group had a significantly higher number of menstrual episodes than the control group (*p* = 0.01). In another study [14], the number of ovarian follicles in the intervention group was significantly decreased (from 17 [SD = 2] to 12 [SD = 2] [*p* < 0.05]), whereas there was no significant difference in the control group. Furthermore, 6 of the 10 patients (60%) in the intervention group showed improved menstrual cycles, while the same was observed in only 3 of the 10 patients (30%) in the control group (*p* < 0.05). Another study [31] reported that four of the six (66.7%) patients in the intervention group displayed ovulation, whereas the same was observed in only one of the six (16.7%) patients in the control group.

The effect of intervention on the reproductive index reported by the studies that did not involve control groups is stated as follows: Among the nine studies that reported the number of patients with improved menstrual cycles after intervention, the proportion of patients with improved cycles ranged from 19.2% to 70%. Ovulation was reported in six studies, and the proportion of patients with ovulation ranged from 35% to 60%. Moreover, the number of ovarian follicles was reported in six studies, among which five studies reported a significant reduction in the number of ovarian follicles after lifestyle intervention compared to the status prior to the intervention.

#### 3.2.2. Metabolic Index

Among the five studies that compared the group that underwent lifestyle intervention programs with the control group [31], one study assessed and reported fasting insulin levels. The intervention group showed a significant decrease in fasting insulin levels after 12 weeks (from 73.6 [SD = 7.01] 57.1 [SD = 11.5]), whereas no significant difference was observed in the control group.

Furthermore, in reference to the variation in parameters before and after lifestyle modification, four of six studies reported a significant reduction in the fasting glucose level after lifestyle intervention compared to the level prior to the intervention. Seven of thirteen studies reported a significant reduction in fasting insulin levels, and four of five studies reported a significant reduction in HOMA-IR scores.

#### 3.2.3. Anthropometric Index

All 25 studies included in the current review reported the effect of lifestyle intervention programs on anthropometric indices. The five studies that compared the group that underwent the lifestyle intervention program with the control group reported that the intervention group showed a statistically significant decrease in BMI (MD: −2.21, 95% CI: −4.22, −0.20, *p* = 0.03, I2 = 0%) and weight (MD: −5.33, 95% CI: −10.28 to −0.38, *p* = 0.03, I2 = 0%), compared to the control group (Figure 3). Furthermore, one study [12] reported that the group that underwent lifestyle modifications had a significantly decreased WC compared to the control group (*p* = 0.029).

In addition, in reference to the variation in parameters before and after lifestyle modification, 7 of 11 studies reported a significant decrease in BMI after lifestyle intervention compared to the situation before intervention, while four studies did not report any statistically significant difference. Moreover, 9 of the 12 studies reported a significant reduction in body weight after lifestyle intervention. All 12 studies that evaluated WC reported a significant decrease after the lifestyle intervention.

### 3.3. Subgroup Analysis

#### 3.3.1. Effectiveness of Lifestyle Modification according to the Type of Intervention

**Figure 4:**
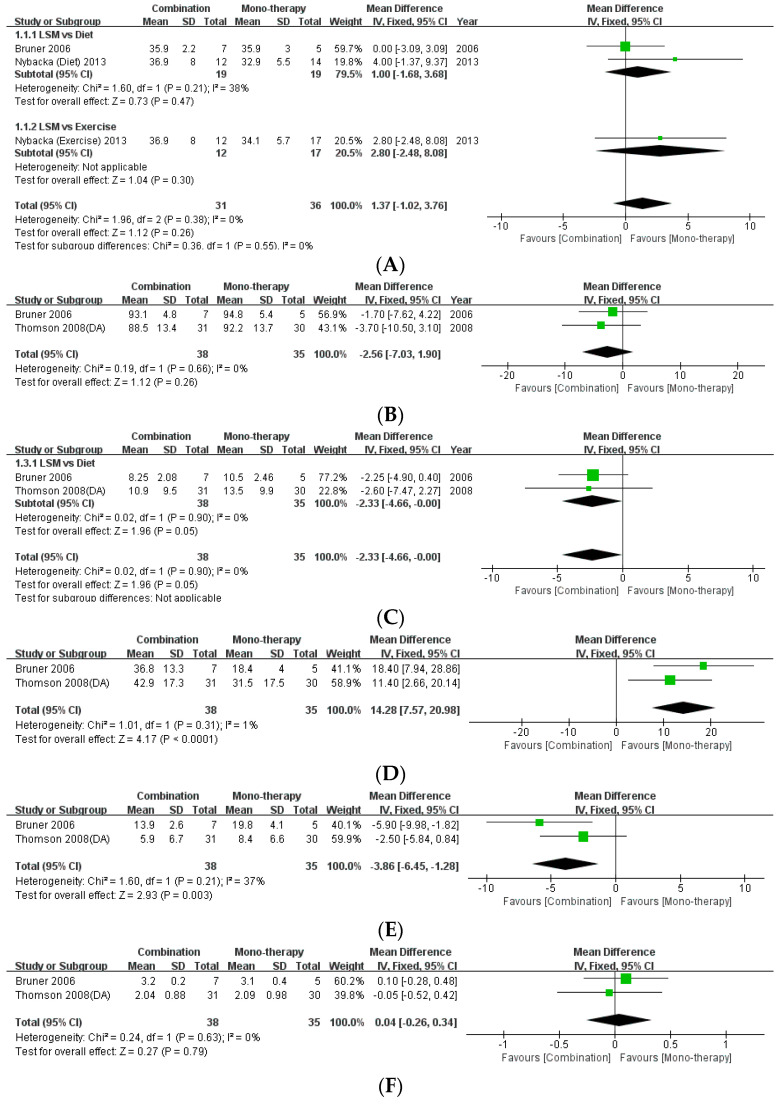
Forest plot for the meta-analysis of combination therapy, compared to monotherapy. (**A**) BMI. (**B**) Waist circumference. (**C**) Fasting insulin level. (**D**) Testosterone level. (**E**) Sex-hormone binding globulin. (**F**) Free androgen index. (CI: confidence interval, SD: standard deviation.)

The present review included three studies that compared the effects of monotherapy (diet/exercise) with combination therapy [16,21,24]. This study performed a meta-analysis of the data pertaining to the anthropometric indices of BMI and WC (Figure 4). There was no statistical difference between monotherapy and combination therapy with regard to BMI (MD: 1.37, 95% CI: −1.02 to 3.76, *p* = 0.26, I2 = 0%) and WC (MD: −2.56, 95% CI: −7.03 to 1.90, *p* = 0.26, I2 = 0%). Moreover, meta-analysis of the data regarding the levels of fasting insulin as a metabolic index revealed that combination therapy effected a statistically significant decrease in fasting insulin levels compared to monotherapy with diet (MD: −2.33, 95% CI: −4.66, −0.00, *p* = 0.05, I2 = 0%). Regarding the difference between monotherapy and combination therapy in the androgenic indices, there was no statistically significant difference in the levels of testosterone (MD: 0.04, 95% CI: −0.26 to −0.34, *p* = 0.79, I2 = 0%). However, combination therapy achieved significant improvement in reference to the levels of SHBG (MD: 14.28, 95% CI: 7.57 to 20.98, *p* < 0.0001, I2 = 1%) and FAI (MD: −3.86, 95% CI: −6.45, −1.28, *p* = 0.003, I2 = 37%) compared to monotherapy.

#### 3.3.2. Effectiveness of Lifestyle Modification according to the Degree of Weight Loss

This review involved three studies that categorized the subjects into groups according to the degree of weight loss and compared the results [17,25,32]. Among the studies, two studies divided the subjects into groups according to the success or failure of weight loss by 5% of body weight [25,32], while one study divided the subjects on the basis of the reduction in respective BMI SD score by 0.2 or more [17].

**Figure 5:**
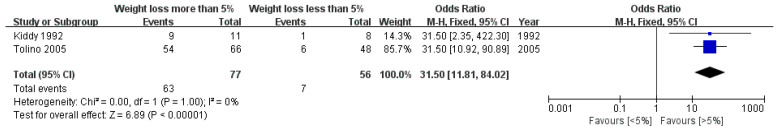
Forest plot for the meta-analysis of improvements in reproductive function according to the degree of weight loss. (CI: confidence interval, SD: standard deviation.)

This study performed a meta-analysis of data pertaining to improvements in reproductive function (Figure 5). The number of patients with improved reproductive function was significantly higher in the group that achieved weight loss of 5% or more compared to the subjects who achieved less than 5% weight loss (OR: 31.50, 95% CI: 11.81 to 84.02, *p* < 0.00001, I2 = 0%).

Moreover, regarding the metabolic index, two studies [25,32] observed a significant reduction in the fasting insulin level in the group that lost more than 5% of body weight, whereas no significant change was observed in the group that lost less than 5% of weight. In addition, one study [17] reported that the group that successfully attained weight loss displayed a tendency toward lower fasting insulin levels (23 to 17), whereas the group that failed to achieve the target weight showed a significant increase in fasting insulin levels (25 to 33, *p* < 0.05).

## 4. Discussion

A systematic review and meta-analysis was performed to confirm the effectiveness of lifestyle modifications in the management of PCOS patients with obesity. To the best of our knowledge, this is the most updated and comprehensive systematic review of this subject. Moreover, this is the first study to report the effects of lifestyle modifications on reproductive function. In addition, the current review performed a subgroup analysis according to the type of intervention and degree of weight loss.

The main finding of the present review is that the group that underwent lifestyle modifications displayed significant improvement in reproductive function compared to the control group. Furthermore, the subgroup analysis revealed that combination therapy with diet and exercise had better effects on metabolic and androgenic parameters than monotherapy. In addition, moderate weight loss by a minimum of 5% of body weight appeared to be effective in improving the metabolic index, that is, fasting insulin levels. The BMI and body weight of the group that underwent lifestyle intervention were significantly lower than those of the control group (BMI: MD −2.21, *p* = 0.03, weight: MD −5.33, *p* = 0.03). These results are concurrent with the results reported by previous studies, which observed that lifestyle modification programs have positive effects on anthropometric indices [7].

### 4.1. Lifestyle Modification Has a Positive Effect on Reproductive Outcomes in PCOS Patients with Obesity

Lifestyle modification plays an important role in the improvement of reproductive outcomes in PCOS patients with obesity. This study performed a meta-analysis of the data pertaining to reproductive function and found that the group that underwent lifestyle modifications had a significantly higher number of patients with improved menstrual cycles compared to the control group (OR: 4.34, *p* = 0.02). Furthermore, the RCTs reported a significant improvement in menstrual episodes or number of ovarian follicles in the group that underwent lifestyle modifications compared to the control group [12,14]. Several therapeutic approaches, such as combined oral contraceptives, insulin sensitizers, anti-androgenic drugs, and assisted reproductive therapy, have been used in the management of PCOS patients who wish to conceive a child. However, these treatments might be associated with the risk of adverse effects [9]. The current results regarding the effect of lifestyle modification alone, without any additional treatment, on the reproductive index are expected to offer encouragement for patients regarding their chances of conception due to the fact that PCOS patients often have concerns about fertility [33].

### 4.2. Combination Therapy with Diet and Exercise Rather Than Monotherapy

It is necessary to recommend healthy lifestyle modifications, including dietary interventions to reduce caloric intake and regular exercise, for obese women with PCOS. The results of the present meta-analysis confirmed that combination therapy had better effects on the improvement of fasting insulin levels (MD: −2.33, *p* = 0.05), SHBG (MD: 14.28, *p* < 0.0001), and FAI (MD: −3.86, *p* = 0.003) compared to monotherapy. Moreover, there was no significant difference between the two interventions with regard to weight loss, and both resulted in adequate weight loss.

The aforementioned result is concurrent with the updated international evidence-based guidelines that recommend a healthy lifestyle involving a healthy diet and regular physical activity for the management of patients with PCOS [2]. A calorie-restricted diet has been mainly used to achieve weight loss in PCOS patients with obesity, and several studies have confirmed the effects of symptomatic improvement along with weight loss [7]. In addition, resistance exercise improves insulin sensitivity by increasing muscle mass, and aerobic exercise improves glucose disposal by increasing glycogen synthase activity [34,35]. Considering the fact that PCOS is characterized by hyperinsulinemia and exacerbated by abdominal obesity [21], a combination of diet and exercise can be a potentially effective mode of treatment.

Furthermore, this study attempted to perform a subgroup analysis according to the type of intervention. However, the analysis could not be performed owing to the heterogeneity of the interventions employed in the studies. Regarding dietary intervention, several nutrient-restricted diets, such as a low-carbohydrate diet, low-fat diet, and high-protein diet, have been followed by PCOS patients. However, the effect of nutrient-restricted diets has not been confirmed to date, and only the effect of a low-calorie diet has been reported in the literature [12,19,23,26]. Hence, this scenario warrants the development of an optimal intervention for the management of PCOS patients with obesity.

### 4.3. Metabolic Index Improved When Moderate Weight Loss Was Achieved

The results reported by previous studies regarding the effects of lifestyle modifications on the metabolic index have been controversial. In addition, recent reviews have reported that the effect of lifestyle interventions on an oral glucose tolerance test is uncertain [7,36]. Furthermore, in this review, the analysis of results concerning the metabolic index was challenging owing to the lack of a sufficient number of studies. Hence, the current study performed a subgroup analysis according to the improvement in fasting insulin level or lack of the same.

In this review, 7 of 13 studies showed improvement in fasting insulin levels after intervention, and six did not report any improvement. Among the 13 studies, 5 compared the body weight before and after the intervention. Studies that involved subjects who attained weight loss by a minimum of 5% of body weight reported an improvement in insulin levels. Nonetheless, subjects who lost less than 5% of their body weight did not display any significant difference in fasting insulin levels after the intervention. Among the studies that divided the subjects into groups according to the degree of weight loss and compared the results, two studies [25,32] observed a significant reduction in insulin levels in the group that lost more than 5% of body weight. No significant change was observed in the group that lost less than 5% of body weight. Considering the abovementioned results, it is assumed that moderate weight loss (minimum of 5%) might be effective in improving the metabolic index.

Insulin resistance is a major cause of the increased severity of PCOS, and PCOS patients with obesity are relatively more vulnerable compared to PCOS patients with normal body weight [37]. Thus, understanding and verifying the metabolic effects of lifestyle modification among PCOS patients with obesity is important from the perspective of management. Because a meta-analysis was not possible due to the lack of a sufficient number of studies, further studies are required to improve the level of evidence on the subject.

### 4.4. Strengths and Limitations

The present systematic review studied the efficacy of lifestyle modification in the management of obese PCOS patients using the most updated data. Moreover, the current study performed subgroup analyses based on the type of intervention and degree of weight loss in order to identify the ideal intervention in such patients.

The current study has certain limitations. First, the sample size of the studies included in the review was not adequate. The scenario warrants high-quality studies with large sample sizes in order to improve the level of evidence. Second, owing to the heterogeneity concerning the study design, it was difficult to conduct a meta-analysis. Comparative analysis in accordance with the type of intervention requires the establishment of a standardized study design. Finally, further studies that include the assessment of pregnancy and ovulation rates as reproductive functions are required to confirm the effect of lifestyle modification among patients with PCOS.

## 5. Conclusions

This review identified evidence supporting the effectiveness of lifestyle modifications in PCOS patients with obesity. Lifestyle modification as a first-line treatment of obese women with PCOS may effect outcomes, and accompanying moderate weight loss is also expected to improve the metabolic index. Lifestyle modification using combination therapy is a promising therapeutic approach that can be employed in the management of PCOS patients with obesity.

## Figures and Tables

**Table 1:** Characteristics of the studies included in the present review.

Name and Year	Country	Study Design	Inclusion Criteria		Intervention		N	Mean Age
			Obesity	PCOS	Type	Group		
Cochrane 2021 [8]	England	Clinical trial	BMI > 28.6	Not specified	Exercise	Exercise	11	30.1 ± 4.6
						Control	4	37.5 ± 4.0
Oberg 2019 [9]	Sweden	RCT	BMI ≥ 27	Rotterdam	LSM	LSM	30	31.0 ± 5.1
						Control	27	29.9 ± 5.7
Kirubamani 2018 [10]	India	One group (pre-post)	BMI ≥ 24.9, BMI < 29.9	Clinical features and USG (not specified)	Exercise	Exercise	50	16–35 (Range)
Deepthi 2017 [11]	Malaysia	One group (pre-post)	BMI ≥ 24.9, BMI < 29.9	USG (not specified)	Exercise	Exercise	30	18–25 (Range)
Marzouk 2015 [12]	Egypt	RCT	BMI > 30	Clinical features	Diet	Diet	30	19.3 ± 1.3
						Control	30	20.1 ± 1.8
Mahoney 2014 [13]	USA	One group (pre-post)	BMI > 27	Rotterdam	LSM	LSM	12	32.0 ± 5.3
Gaeini 2014 [14]	Iran	RCT	BMI > 25	Not specified	Exercise	Exercise	10	23.6 ± 5.0
						Control	10	
Roessler 2013 [15]	Denmark	One group (pre-post)	BMI ≥ 25, BMI ≤ 40	Rotterdam	Exercise	Exercise	17	31.6
Nybacka 2013 [16]	Sweden	RCT	BMI > 27	Rotterdam	LSM	Diet	19	29.9 ± 5.5
						Exercise	19	31.3 ± 4.8
						LSM	19	31.8 ± 4.9
Lass 2012 [17]	Germany	Prospective (longitudinal)	97th BMI percentile by age for German adolescents	National Institutes of Health	LSM	LSM (weight loss+)	26	31.8 ± 4.9
						LSM (weight loss-)	33	15.0 ± 0.7
Redman 2011 [18]	USA	Prospective	BMI ≥ 25	Not specified	Exercise	Exercise	8	18–30 (range)
Ornstein 2011 [19]	USA	Clinical trial	85th BMI percentile by age	Clinical features	Diet	Diet (LC)	12	15.8 ± 2.2
						Diet (LF)	12	
Thomson 2009 [20]	Australia	Prospective	Mean BMI 36.2 ± 0.8	Rotterdam	Diet	Diet (R)	52	29.2 ± 0.9
						Diet (R-)		
Thomson 2008 [21]	Australia	Clinical trial	BMI ≥ 25	Rotterdam	LSM	Diet	30	29.3 ± 0.7
						LSM (DA)	31	
						LSM (DC)	33	
Palomba 2008 [22]	Italy	Clinical trial	BMI > 30, BMI ≤ 35	Rotterdam and National Institutes of Health	LSM	Diet	20	26.8 ± 5.1
						Exercise	20	25.8 ± 4.5
Moran 2006 [23]	Australia	RCT	BMI ≥ 25	Rotterdam	Diet	Diet (CC)	22	32.1 ± 5.5
						Diet (FC)	21	33.2 ± 4.8
Bruner 2006 [24]	Canada	RCT	BMI > 27	Rotterdam	LSM	Diet	5	28.4 ± 2.7
						LSM	7	32.3 ± 1.0
Tolino 2005 [25]	Italy	Prospective	BMI ≥ 25	Clinical features, polycystic ovaries by USG	Diet	Diet (weight loss > 5%)	78	NR
						Diet (weight loss < 5%)	17	
Stamets 2004 [26]	USA	RCT	BMI ≥ 25	Clinical features	Diet	Diet (HP)	13	29 ± 4
						Diet (HC)	13	26 ± 4
van Dam 2004 [27]	USA	Prospective	BMI > 30	Clinical features	Diet	Diet (R+)	9	30 ± 2.5
						Diet (R−)	6	30 ± 1.8
Crosignani 2003 [28]	Italy	Prospective	BMI ≥ 25	USG (not specified)	LSM	LSM	33	30.7 ± 3.9
Moran 2003 [29]	Australia	Clinical trial	Mean BMI 37.7 ± 1.9 (LP) 37.9 ± 1.6 (HP)	Clinical features	LSM	LSM (LP)	14	33 ± 1.2
						LSM (HP)	14	32 ± 1.2
Huber-buchholz 1999 [30]	Australia	Prospective	BMI ≥ 27, BMI ≤ 45	Not specified	LSM	LSM (R+)	7	28.7 ± 0.9
						LSM (R−)	6	28.7 ± 0.9
Guzick 1994 [31]	USA	RCT	130–200% of ideal body weight	Not specified	LSM	LSM	6	32.2 ± 4.9
						Control	6	31.2 ± 3.9
Kiddy 1992 [32]	UK	Prospective	Mean weight 91.5 ± 14.7	Clinical features, polycystic ovaries by USG	Diet	Diet (weight loss > 5%)	24	NR
						Diet (weight loss < 5%)		

BMI: body mass index, CC: carbohydrate-counting, DA: diet and aerobic exercise, DC: diet and combined aerobic-resistance exercise, FC: fat-counting, HC: high carbohydrate, HP: high protein, LC: low carbohydrate, LF: low fat, LP: low protein, LSM: lifestyle modification (diet and exercise), N: number, NR: no response, PCOS: polycystic ovary syndrome, R: response, RCT: randomized controlled trial, UK: United Kingdom, USA: United states of America, USG: ultrasonography.

**Table 2:** Characteristics of the lifestyle modification program.

Name and Year	Type of Intervention	Type of Diet/Exercise	Composition of Diet/Exercise Program	Duration, Frequency, Time
Cochrane 2021	Exercise	Aerobic	Aerobic, aquarobics, gym session	12 weeks, 2 days/week, 60 min/day
Oberg 2019	LSM	Counseling	Personalized coaching for physical activity and diet	4 months
Kirubamani 2018	Exercise	Aerobic	Walking, running (treadmill): warm up: 5 min, exercise: 35 min, cool down: 5 min	16 weeks, 5 days/week, 45 min/day
Deepthi 2017	Exercise	Aerobic	Walking, running (treadmill): warm up: 5 min, exercise: 35 min, cool down: 5 min	8 weeks, 3 days/week, 45 min/day
Marzouk 2015	Diet	Calorie reduction	500 kcal deficit/day	6 months
		Diet counseling	Increase in low-GI foods, decrease in high saturated fats Intake of multivitamin supplements	
Mahoney 2014	LSM	Calorie reduction	Not specified	12 weeks
		Diet counseling	Increase in low-GI foods, decrease in saturated fats	
		Aerobic	Walking, cycling, aerobics	12 weeks, 3–5 days/week, 30–60 min/day
		Resistance	Major muscle strength training (not specified)	12 weeks, 2–3 days/week, 30–60 min/day
Gaeini 2014	Exercise	Aerobic	Running	12 weeks, 3 days/week, 25–30 min/day
Roessler 2013	Exercise	Aerobic	Walking, running	8 weeks, 1 day/week, 25–45 min/day
			Cycling	8 weeks, 2 days/week, 35–55 min/day
Nybacka 2013	LSM	Calorie reduction	Reduction of 600 calories, compared to the prior intake	4 months
		Diet composition	Carbohydrate (55–60%), fat (25–30%), protein (10–15%)	
		Aerobic	Walking, jogging, aerobics, swimming	4 months, 2–3 days/week, 45–60 min/day
		Resistance	Muscle strength training (not specified)	
Lass 2012	LSM	Diet composition	Carbohydrate (55% with 5% sugar), fat (30%), protein (15%)	3 months
		Aerobic	Dancing, ball games, jogging, trampoline jumping	1 year, 1 day/week
Redman 2011	Exercise	Aerobic	Aerobic exercise	16 weeks, 5 days/week
Ornstein 2011	Diet	Calorie reduction	Not specified	12 weeks
		Diet counseling	Increase in low-GI foods Intake of multivitamin supplements	
		Diet composition 1	Low carbohydrate: carbohydrate 40 g/day	
		Diet composition 2	Low fat: fat less than 50 g/day	
Thomson 2009	Diet	Calorie reduction	6000 kJ/day	20 weeks
Thomson 2008	LSM	Calorie reduction	5000–6000 kJ/day	20 weeks
		Diet composition	High protein: carbohydrate (40%), fat (30%, saturated fat < 8%), protein (30%)	
		Aerobic	Walking, jogging	20 weeks, 3 days/week, 20–45 min/day
		Resistance	Bench press, lag pull down, leg press, knee extension, and sit-ups	20 weeks, 2 days/week
Palomba 2008	LSM	Calorie reduction	800 kcal deficit/day	24 weeks
		Diet composition	High protein: carbohydrate (45%), fat (20%), protein (35%)	
		Diet counseling	Taking the multivitamin/mineral supplement	
		Aerobic	Cycling: warm up: 5 min, exercise: 35 min, cool down: 5 min	24 weeks, 3 days/week, 45 min/day
Moran 2006	Diet	Calorie reduction	Meal replacement: 2 meals/day of a meal termed Slimfast	0–8th weeks
		Diet composition 1	Low carbohydrate: carbohydrate up to 120 g/day	9th–32nd weeks
		Diet composition 2	Low fat: fat up to 50 g/day	
Bruner 2006	LSM	Diet counseling	Canada’s food guide to healthy eating	12 weeks
		Aerobic	Walking and/or cycling: warm up: 10 min, exercise: 30 min	12 weeks, 3 days/week, up to 90 min/day
		Resistance	biceps curl, lag pull down, leg curl, leg extension, shoulder press, chest press, hip abduction, hip adduction, hip flexion, hip extension, and back extension	
Tolino 2005	Diet	Calorie reduction	1000 kcal/day (patients with BMI ≤ 30) 500 kcal/day (patients with BMI > 30, initial 4 weeks)	7 months
		Diet composition	Low fat (not specified)	
Stamets 2004	Diet	Calorie reduction	1000 kcal deficit/day	1 month
		Diet composition 1	High protein: carbohydrate (55%), fat (30%), protein (30%)	
		Diet composition 2	High carbohydrate: carbohydrate (45%), fat (20%), protein (15%)	
van Dam 2004	Diet	Calorie reduction	Meal replacement: 470 kcal/day of a meal named Modifast	7 days
		Diet composition	Carbohydrate (42%), fat (15%), protein (43%)	
Crosignani 2003	LSM	Calorie reduction	1200 kcal/day	6 months
		Diet Composition	Carbohydrate (55%), fat (25%), protein (20%), fiber (30 g/week)	
		Aerobic	Aerobic exercise	6 months, 1–2 days/week
Moran 2003	Diet	Calorie reduction	6000 kJ/day	16 weeks
		Diet composition 1	Low protein: carbohydrate (55%), fat (30%), protein (15%)	
		Diet composition 2	High protein: carbohydrate (40%), fat (30%), protein (30%)	
Huber-buchholz (1999)	LSM	Not specified	Not specified	Not specified
Guzick 1994	LSM	Calorie reduction	Meal replacement: 400 kcal/day of a meal named Optifast	0–8th weeks
			4200–5040 kJ/day	9th–12th weeks
		Aerobic	Walking	12 weeks, 5 days/week
Kiddy 1992	Diet	Calorie reduction	1000 kcal/day (patients with BMI ≤ 30) 330 kcal/day (patients with BMI > 30, initial 4 weeks)	7 months
		Diet composition	Low fat (not specified)	

BMI: Body mass index, GI: glycemic index, LSM: lifestyle modification (diet and exercise).

**Table 3:** Reproductive effects of the lifestyle modification program.

Name and Year	Follow-Up	Reproductive Index	Groups	Before	After
Oberg 2019	4 months	No. of patients with improvement in menstrual cycle	LSM		20/34 (58.8%)
			Control		8/34 (23.5%) ^†^
		No. of patients with ovulation	LSM		7/34 (20.6%)
			Control		7/34 (20.6%)
	12 months	No. of patients who conceive	All		11/68 (16.2%)
Kirubamani 2018	16 weeks	No. of patients with irregular menstrual cycle	Exercise	38.6 ± 6.7	14.4 ± 1.5 *
		No. of patients with ovulation	Exercise	13.1 ± 1.5	30.3 ± 3.7 *
		Number of ovarian follicles	Exercise	10–12	6–8 *
		Diameter ovarian follicle	Exercise	7.4	7.1
		Ovarian volume	Exercise	11.2	9.2 *
Deepthi 2017	8 weeks	No. of patients with regular menstrual cycle	Exercise		28/30 (93.3%)
		Number of ovarian follicles	Exercise	16.7	14.2 *
Marzouk 2015	6 months	No. of menstrual episodes	Diet	2.4 ± 1.6	3.1 ± 1.2 ^†^
			Control	2.2 ± 1.3	2.3 ± 1.3
Mahoney 2014	12 weeks	No. of patients with improvement in menstrual cycle	LSM		2/8 (25%)
Gaeini 2014	12 weeks	Number of ovarian follicles (left)	Exercise	17 ± 2	12 ± 2 *
			Control	17 ± 5	18 ± 3
		Number of ovarian follicles (right)	Exercise	16 ± 4	14 ± 2 *
			Control	18 ± 4	18 ± 3
		No. of patients with improvement in menstrual cycle	Exercise		6/10 *
			Control		3/10
Roessler 2013	16 weeks	No. of patients with regular menstrual cycle	Exercise		4/17 (23.5%)
		Ovarian volume	Exercise	12.7 ± 1.2	12.2 ± 1.2
Nybacka 2013	4 months	No. of patients with improvement in menstrual cycle	LSM		30/43 (70%)
		No. of patients with ovulation	LSM		15/43 (35%)
		Number of ovarian follicles (mean)	Diet	12.4 ± 3.9	9.4 ± 2.4 *
			Exercise	13.2 ± 4.6	10.5 ± 3.2 *
			LSM	12.8 ± 4.7	10.1 ± 3.4 *
		Ovarian volume	Diet	9.6	7.7
			Exercise	9.6	10.9
			LSM	8.8	12.4
Lass 2012	1 year	No. of patients with irregular menstrual cycle (amenorrhea)	Success in weight loss	18/26 (69%)	7/26 (27%) *
			Failure in weight loss	20/33 (61%)	18/33 (55%)
		No. of patients with irregular menstrual cycle (oligomenorrhea)	Success in weight loss	8/26 (31%)	3/26 (12%)
			Failure in weight loss	14/33 (39%)	12/33 (36%)
Redman 2011	16 weeks	Number of ovarian follicles	LSM		−15 ± 5 *
		Number of follicles in polycystic ovary	LSM		−15 ± 6 *
		Ovarian volume	LSM		−6 ± 4
Ornstein 2011	12 weeks	No. of patients with menstrual cycle	Diet		12/16 (75%)
		No. of patients with regular menstrual cycle	Diet		8/16 (50%)
		Average no. of bleeding episodes	Diet	0.6 ± 0.6	1.6 ± 1.3 *
Marsh 2010	1 year	No. of patients with improvement in menstrual cycle	Diet 1		NR/NR (95%) ^†^
			Diet 2		NR/NR (63%)
Thomson 2009	20 weeks	No. of patients with improvement in ovulation	Diet		22/52 (42.3%)
		No. of patients with improvement in menstrual cycle	Diet		10/52 (19.2%)
Thomson 2008	20 weeks	No. of patients with improvement in ovulation	Diet		6/12 (50%)
			LSM (DA)		3/6 (50%)
			LSM (DC)		3/7 (42.9%)
		No. of patients with improvement in menstrual cycle	Diet		3/14 (21.4%)
			LSM (DA)		9/21 (42.9%)
			LSM (DC)		8/18 (44.4%)
Palomba 2008	24 weeks	Frequency of menstruation (no. observed menses/no. expected cycles)	Diet		18/118 (15.3%)
			Exercise		28/107 (26.2%) ^†^
		Ovulation rate (no. ovulatory cycles/no. observed cycles)	Diet		18/119 (15.1%)
			Exercise		28/113 (24.8%) ^†^
		Pregnancy rate (no. pregnancy/no. observed cycles)	Diet		2/119 (1.7%)
			Exercise		7/113 (6.2%)
		Cumulative ovulation rate (no. of patients with ovulation)	Diet		5/20 (25.0%)
			Exercise		13/20 (65.0%) ^†^
		Cumulative pregnancy rate (no. of patients who conceived)	Diet		2/20 (10.0%)
			Exercise		7/20 (35.0%)
Moran 2006	32 weeks	No. of patients with improvement in menstrual cycle	Diet		16/28 (57.1%)
Bruner 2006	12 weeks	No. of patients who conceived	LSM		1/12 (8.3%)
		Number of ovarian follicles (left)	Diet	33 ± 4	39 ± 7
			LSM	35 ± 5	39 ± 6
		Number of ovarian follicles (right)	Diet	47 ± 8	46 ± 8
			LSM	49 ± 7	44 ± 5
Tolino 2005	7 months	No. of patients with improvement in ovulation	Diet (>5% loss)		6/66 (9.09%)
		No. of patients with improvement in menstrual cycle	Diet (>5% loss)		18/66 (27.3%)
		No. of patients who conceived	Diet (>5% loss)		30/66 (45.5%)
van Dam 2004	until 10% weight loss	No. of patients with improvement in ovulation	Diet		9/15 (60%)
Crosignani 2003	1 year	No. of patients who conceived	LSM		10/33 (30%)
	6 months	No. of patients with improvement in menstrual cycle	LSM		18/33 (54.5%)
		Ovarian follicle number	5% loss	23.5 ± 11.5	19.9 ± 9.9 *
			10% loss	23.5 ± 11.5	18.3 ± 7.5 *
Moran 2003		No. of patients with improvement in menstrual cycle	Diet		11/25 (44.0%)
Huber-buchholz (1999)	6 months	No. of patients who conceived	LSM		2/15 (13.3%)
		No. of patients with ovulation	LSM		9/15 (60%)
Guzick 1994	12 weeks	No. of patients with ovulation	LSM		4/6 (66.7%)
			Control		1/6 (16.7%)
Kiddy 1992	7 months	No. of patients with improvement in reproductive function	Diet (>5% loss)		9/11 (81.8%) ^†^
			Diet (<5% loss)		1/8 (12.5%)

* *p* < 0.05, compared to the status before intervention. ^†^ *p* < 0.05 versus other groups. DA: Diet and aerobic exercise, DC: diet and combined aerobic-resistance exercise, LSM: lifestyle modification (diet and exercise).

## Data Availability

Not applicable.

## Supplementary Materials

The following supporting information can be downloaded at: https://www.mdpi.com/article/10.3390/life12020308/s1, Table S1: PRISMA 2020 checklist; List S1: Study protocol.

## References

[1] Patten R., Boyle R.A., Moholdt T., Kiel I., Hopkins W.G., Harrison C.L., Stepto N.K. (2020). Exercise interventions in polycystic ovary syndrome: A systematic review and meta-analysis. Front. Physiol..

[2] Teede H.J., Misso M.L., Costello M.F., Dokras A., Laven J., Moran L., Piltonen T., Norman R.J. (2018). Recommendations from the international evidence-based guideline for the assessment and management of polycystic ovary syndrome. Hum. Reprod..

[3] Hoeger K.M., Dokras A., Piltonen T. (2020). Update on PCOS: Consequences, challenges, and guiding treatment. J. Clin. Endocrinol. Metab..

[4] Acién P., Quereda F., Matallıín P., Villarroya E., López-Fernández J.A., Acién M., Mauri M., Alfayate R. (1999). Insulin, androgens, and obesity in women with and without polycystic ovary syndrome: A heterogeneous group of disorders. Fertil. Steril..

[5] Teede H.J., Joham A.E., Paul E., Moran L., Loxton D., Jolley D., Lombard C. (2013). Longitudinal weight gain in women identified with polycystic ovary syndrome: Results of an observational study in young women. Obesity.

[6] Lim S., Davies M., Norman R., Moran L. (2012). Overweight, obesity and central obesity in women with polycystic ovary syndrome: A systematic review and meta-analysis. Hum. Reprod. Update.

[7] Lim S.S., Hutchison S.K., Van Ryswyk E., Norman R.J., Teede H.J., Moran L.J. (2019). Lifestyle changes in women with polycystic ovary syndrome. Cochrane Database Syst. Rev..

[8] Cochrane T., Tengku-Kamalden T.F., Davey R., Dev R.D.O. (2021). Effect of Exercise and Weight Loss in Polycystic Ovarian Syndrome among Obese Women. Pertanika J. Soc. Sci. Hum..

[9] Oberg E., Gidlöf S., Jakson I., Mitsell M., Egnell P.T., Hirschberg A.L. (2019). Improved menstrual function in obese women with polycystic ovary syndrome after behavioural modification intervention—A randomized controlled trial. Clin. Endocrinol..

[10] Kirubamani H., Abraham M. (2018). Effect of aerobic exercise (self-help strategy) on the common endocrine problem (PCOS) in late adolescent & young women & impact on their quality of life. Int. J. Res. Pharm. Sci..

[11] Deepthi G., Sankarakumaran P., Jerome A., Kalirathinam D., Raj N.B., Us M.R. (2017). Effect of aerobic exercise in improving the quality of life in polycystic ovarian disease. Res. J. Pharm. Technol..

[12] Marzouk T.M., Ahmed W.A.S. (2015). Effect of dietary weight loss on menstrual regularity in obese young adult women with polycystic ovary syndrome. J. Pediatr. Adolesc. Gynecol..

[13] Mahoney D. (2014). Lifestyle modification intervention among infertile overweight and obese women with polycystic ovary syndrome. J. Am. Assoc. Nurse Pract..

[14] Gaeini A., Satarifard S., Mohamadi F., Choobineh S. (2014). The effect of 12 weeks aerobic exercise on DHEAso4, 17OH-Progestron concentrations, number of follicles and menstrual condition of women with PCOS. Hormozgan Med. J..

[15] Roessler K.K., Birkebaek C., Ravn P., Andersen M.S., Glintborg D. (2013). Effects of exercise and group counselling on body composition and VO 2max in overweight women with polycystic ovary syndrome. Acta Obstet. Gynecol. Scand..

[16] Nybacka Å., Carlström K., Fabri F., Hellström P.M., Hirschberg A.L. (2013). Serum antimüllerian hormone in response to dietary management and/or physical exercise in overweight/obese women with polycystic ovary syndrome: Secondary analysis of a randomized controlled trial. Fertil. Steril..

[17] Lass N., Kleber M., Winkel K., Wunsch R., Reinehr T. (2011). Effect of lifestyle intervention on features of polycystic ovarian syndrome, metabolic syndrome, and intima-media thickness in obese adolescent girls. J. Clin. Endocrinol. Metab..

[18] Redman L.M., Elkind-Hirsch K., Ravussin E. (2011). Aerobic exercise in women with polycystic ovary syndrome improves ovarian morphology independent of changes in body composition. Fertil. Steril..

[19] Ornstein R.M., Copperman N.M., Jacobson M.S. (2011). Effect of weight loss on menstrual function in adolescents with polycystic ovary syndrome. J. Pediatr. Adolesc. Gynecol..

[20] Thomson R., Buckley J., Moran L., Noakes M., Clifton P., Norman R., Brinkworth G. (2009). The effect of weight loss on anti-Mullerian hormone levels in overweight and obese women with polycystic ovary syndrome and reproductive impairment. Hum. Reprod..

[21] Thomson R., Buckley J., Noakes M., Clifton P., Norman R., Brinkworth G. (2008). The effect of a hypocaloric diet with and without exercise training on body composition, cardiometabolic risk profile, and reproductive function in overweight and obese women with polycystic ovary syndrome. J. Clin. Endocrinol. Metab..

[22] Palomba S., Giallauria F., Falbo A., Russo T., Oppedisano R., Tolino A., Colao A., Vigorito C., Zullo F., Orio F. (2007). Structured exercise training programme versus hypocaloric hyperproteic diet in obese polycystic ovary syndrome patients with anovulatory infertility: A 24-week pilot study. Hum. Reprod..

[23] Moran L.J., Noakes M., Clifton P., Wittert G.A., Williams G., Norman R. (2006). Short-term meal replacements followed by dietary macronutrient restriction enhance weight loss in polycystic ovary syndrome. Am. J. Clin. Nutr..

[24] Bruner B., Chad K., Chizen D. (2006). Effects of exercise and nutritional counseling in women with polycystic ovary syndrome. Appl. Physiol. Nutr. Metab..

[25] Tolino A., Gambardella V., Caccavale C., D’Ettore A., Giannotti F., D’Antò V., De Falco C. (2005). Evaluation of ovarian functionality after a dietary treatment in obese women with polycystic ovary syndrome. Eur. J. Obstet. Gynecol. Reprod. Biol..

[26] Stamets K., Taylor D.S., Kunselman A., Demers L.M., Pelkman C.L., Legro R.S. (2004). A randomized trial of the effects of two types of short-term hypocaloric diets on weight loss in women with polycystic ovary syndrome. Fertil. Steril..

[27] Van Dam E.W., Roelfsema F., Veldhuis J.D., Hogendoorn S., Westenberg J., Helmerhorst F.M., Frolich M., Krans H.M.J., Meinders E., Pijl H. (2004). Retention of estradiol negative feedback relationship to LH predicts ovulation in response to caloric restriction and weight loss in obese patients with polycystic ovary syndrome. Am. J. Physiol. Endocrinol. Metab..

[28] Crosignani P.G., Colombo M., Vegetti W., Somigliana E., Gessati A., Ragni G. (2003). Overweight and obese anovulatory patients with polycystic ovaries: Parallel improvements in anthropometric indices, ovarian physiology and fertility rate induced by diet. Hum. Reprod..

[29] Moran L.J., Noakes M., Clifton P.M., Tomlinson L., Norman R. (2003). Dietary composition in restoring reproductive and metabolic physiology in overweight women with polycystic ovary syndrome. J. Clin. Endocrinol. Metab..

[30] Huber-Buchholz M.-M., Carey D., Norman R. (1999). Restoration of reproductive potential by lifestyle modification in obese polycystic ovary syndrome: Role of insulin sensitivity and luteinizing hormone. J. Clin. Endocrinol. Metab..

[31] Guzick D.S., Wing R., Smith D., Berga S.L., Winters S.J. (1994). Endocrine consequences of weight loss in obese, hyperandrogenic, anovulatory women. Fertil. Steril..

[32] Kiddy D.S., Hamilton-Fairley D., Bush A., Short F., Anyaoku V., Reed M.J., Franks S. (1992). Improvement in endocrine and ovarian function during dietary treatment of obese women with polycystic ovary syndrome. Clin. Endocrinol..

[33] Holton S., Hammarberg K., Johnson L. (2018). Fertility concerns and related information needs and preferences of women with PCOS. Hum. Reprod. Open.

[34] Cauza E., Hanusch-Enserer U., Strasser B., Ludvik B., Metz-Schimmerl S., Pacini G., Wagner O., Georg P., Prager R., Kostner K. (2005). The relative benefits of endurance and strength training on the metabolic factors and muscle function of people with type 2 diabetes mellitus. Arch. Phys. Med. Rehabil..

[35] Ivy J.L. (1997). Role of exercise training in the prevention and treatment of insulin resistance and non-insulin-dependent diabetes mellitus. Sports Med..

[36] Domecq J.P., Prutsky G., Mullan R.J., Hazem A., Sundaresh V., Elamin M.B., Phung O.J., Wang A., Hoeger K., Pasquali R. (2013). Lifestyle modification programs in polycystic ovary syndrome: Systematic review and meta-analysis. J. Clin. Endocrinol. Metab..

[37] Legro R.S. (2012). Obesity and PCOS: Implications for diagnosis and treatment. Seminars in Reproductive Medicine.

