# Supplementary material for: Effectiveness of Lifestyle Modification in Polycystic Ovary Syndrome Patients with Obesity: A Systematic Review and Meta-Analysis
Source: Life (Basel). 2022 Feb 18;12(2):308. doi: 10.3390/life12020308 (PMC8876590; doi:10.3390/life12020308)
Supplement: Supplementary file 1 [file life-12-00308-s001.zip › life-1565884-supplementary List S1.pdf]

Supplementary Materials (List S1: Study protocol)

Review question

To confirm the effectiveness of lifestyle modification programs in the management of obese PCOS patients with obesity

Searches

Databases: Cochrane library, Ovid-Embase, Ovid-Medline

Search term: Keywords and mesh terms of "exp body mass index/," "exp overweight/," "exp obesity/," "BMI," "exp infertility/," "exp anovulation/," and "exp polycystic ovary syndrome/." The terms pertaining to intervention included: "lifestyle modification," "lifestyle intervention," "lifestyle change," "lifestyle program," "exp diet/," "exp exercise/," and "exp weight loss/."

Types of study to be included

(a) studies that involved patients diagnosed with polycystic ovary syndrome and obesity
(b) studies that involved a lifestyle intervention program (diet and/or exercise program)
(c) studies that assessed more than one variable of interest (reproductive, anthropometric, androgenic, and metabolic indices)
(d) studies that were not review articles, abstracts, conference posters, articles written in languages other than English

Participants/population

Participants who were diagnosed in polycystic ovary syndrome and obesity

Intervention(s), exposure(s)

Lifestyle modification program (diet and/or exercise program)
(Studies with concurrent drug use will be excluded)

Comparator(s)/control

Not applicable

Main outcome(s)

(a) reproductive index: number of patients with regular/irregular menstrual cycle, number of patients with improvement in menstrual cycle, ovarian volume, and number of ovarian follicles
(b) anthropometric index: weight, body mass index, and waist circumference
(c) metabolic index: fasting glucose level, fasting insulin level, and homeostatic model assessment for insulin resistance

(d) androgenic index: testosterone level, sex-hormone binding globulin (SHBG), and free androgen index

Additional outcome(s)

None

Data extraction (selection and coding)

All the reviewers will perform data extraction using a prearranged data extraction form and double-checked the same.
(a) Characteristics pertaining to the included participants (age, definition of obesity, and criteria used to diagnose PCOS)
(b) Type of intervention employed by the studies (specific components, duration, frequency, time of day)

Risk of bias (quality) assessment

The tools: the Cochrane Risk of Bias tool (RoB) and the Risk of Bias Assessment Tool for Non-randomized Studies (RoBANS)

Strategy for data synthesis

Data management and meta-analysis will be performed using the RevMan program (Review Manager 5.4) The current study will compute the combined estimate of the odds ratios (ORs) pertaining to two groups using the Mantel–Haenszel method to assess dichotomous variables. The effect measures of continuous variables will be estimated as mean difference (MD) and 95% confidence intervals (CI) using the inverse variance method. The chi-squared test will be used to assess statistical heterogeneity among studies, and the significance will be set at $p<0.10$. Heterogeneity will be quantified using the I^2 statistic. A p-value less than 0.05 was considered to be statistically significant.

Analysis of subgroups or subsets

(a) Effectiveness of lifestyle modification according to the type of intervention (mono-therapy versus combined therapy)
(b) Effectiveness of lifestyle modification according to the degree of weight loss (not specific)

Organisational affiliation of the review

Gachon university (https://www.gachon.ac.kr/eng/index.do#this)

Review team members and their organisational affiliations

Ms. Chan Hee Kim, Sejong hospital

Professor. SeonHeui Lee, Gachon university

Type and method of review

Systematic review and meta-analysis

Anticipated or actual start date

August, 2020

Anticipated completion date

December, 2021

Funding sources/sponsors

The National Research Foundation of Korea (NRF) funded by the Korean government (MSIT) (No. 2020R1F1A1073141).

Conflicts of interest

syndrome The authors declare no conflict of interest.

Language

English

Country

South Korea
